# Feasible Use of Recycled Concrete Aggregates with Alumina Waste in Road Construction

**DOI:** 10.3390/ma14061466

**Published:** 2021-03-17

**Authors:** Manuel Cabrera, Mónica López-Alonso, Laura Garach, Javier Alegre, Javier Ordoñez, Francisco Agrela

**Affiliations:** 1 Area of Construction Engineering, University of Cordoba, 14071 Cordoba, Spain; manuel.cabrera@uco.es; 2 Civil Engineering School, University of Granada, 18010 Granada, Spain; mlopeza@ugr.es (M.L.-A.); lgarach@ugr.es (L.G.); fjalegre@ugr.es (J.A.); javiord@ugr.es (J.O.)

**Keywords:** recycled aggregates, recycled alumina waste, civil infrastructures, road section, real scale

## Abstract

The management of different industrial by-products, such as recycled aggregates from construction and demolition waste and alumina by-products, as well as the reduction of landfill deposits by incorporating these products in a second life cycle, were the focus of this work. The aim of this study was to demonstrate the technical viability of using these waste and by-product as a material for road pavement base layers. For this purpose, a real-scale application was carried out, and the behavior of three types of materials, applied on a section of an experimental road under real vehicle traffic conditions, was studied and compared. Three materials were used in these sections applied in the road sub-bases. First, a control material composed of a type of artificial gravel was used to be compared with the rest of materials; the second material was composed of recycled aggregates, and the third was composed of a mix of recycled aggregates and alumina waste. The results concluded that the effectiveness of the sections built using recycled aggregates and alumina waste was very positive and similar those constructed using natural aggregates.

## 1. Introduction

Almost all industrial and human activities produce waste, and its increasing accumulation is the cause of serious environmental and economic issues around the world. The total waste generated in the European Union amounted to 2.51 billion tons [1].

The use of recycled aggregates (RA) made from construction and demolition waste (CDW) has increased considerably in civil engineering. In the recent past, extensive research works have been carried out to evaluate different properties of using RA [2,3,4,5,6].

Some studies have indicated that recycled concrete aggregates (RCA) could successfully be used as a substitute of natural aggregates to produce concrete [7,8,9], meeting the required performance of structural and non-structural concrete [10,11].

Several researchers have studied the manufacture of mortar, although scientific and technical studies on the use of RA from CDW in the manufacture of mortar are scarce and mainly limited to the use of RCA [12,13,14,15].

Poon [16] conducted a thorough investigation of the feasibility of using RCA in the production of paving stones. They concluded that the replacement of coarse and fine natural aggregates with recycled aggregates concrete can reach 50% without registering a reduction in the compressive strength of the paving blocks.

The possibility of using RA of CDW in road construction has been studied by many researchers [17,18,19,20,21,22,23,24,25,26,27,28], who evaluated the feasibility of using RA as a granular material in the structural layer of pavement.

Molenaar [17] conducted an exhaustive study of RCA as unbound material for road bases, reporting that the most influential factor regarding mechanical properties is the degree of compaction of the material, an easy property to implement and control compared to other properties. Herrador [20] incorporated RA in the construction of two road sections to study their behavior in real conditions. They reported that it is feasible to use RA in subbase layers instead of natural aggregate because the recycled material showed appropriate characteristics.

Jimenez [21] reported the possible use of unbound recycled aggregates from selected CDW in unpaved rural roads, obtaining high values of load capacity and structural stability.

Perez [22] studied the efficiency of incorporating RCA treated with cement in the construction of a road in Malaga (Spain). The natural coarse aggregate was replaced by RCA with a particular size similar to that of the natural aggregate. They reported that it is possible to use RCA if there is good production and execution control. Del Rey [23] used recycled mixed aggregate (RMA) in structural layers of an unpaved rural road with low traffic intensity with positive results. Tavira [26,27] tested the use of RA in unbound layers and evaluated deflections and surface roughness for a period of 7 years to study the behavior of these recycled materials in the long term. The layers manufactured with RA showed good mechanical behavior. Li [25] studied the deformations, California bearing ratio (CBR), and settlements in a section of road built with RA, and testing showed a greater load capacity than that of the natural aggregate.

On the other hand, the need to conserve and preserve the environment begins by reducing solid waste produced by industry and recycling the maximum volume of material. The alumina waste (AW) is an example of waste that needs to be studied and valued to include it as a raw material in construction materials. Manfredi [29] defined aluminum slag as a set of free metals and non-metallic compounds produced by the aluminum industry.

Sua-Iam [30] studied alumina residues as a possible cementitious material by incorporating different proportions of AW as a replacement of fine natural aggregate in self-compacting concretes. The results showed that mixtures that incorporated 75% AW improved the short- and long-term mechanics, mainly due to pozzolanic reactions. AW has also been used as a cement replacement in the manufacturing of conventional concrete. Elinwa [31] reported that with a 10% cement replacement, the compressive and flexural strength was similar to that of conventional control concrete.

Lopez-Alonso [32] studied at laboratory scale the use of AW with RA for possible use in road layers, reporting that there was an increase in compressive strength at 28 days.

At present, there has been no real-scale study of the application of RCA in combination with AW in the construction of road structural layers.

The objective of this research was to evaluate the behavior of RCA and RCA mixed with AW in the structural layers of a highway. The results were compared with those obtained using a control material, artificial gravel (AG). For this, the construction of an experimental highway with three different sections was carried out: one made with AG, another with RCA, and a third section with RCA + AW.

## 2. Design of the Experimental Road Sections

**Figure 1:**
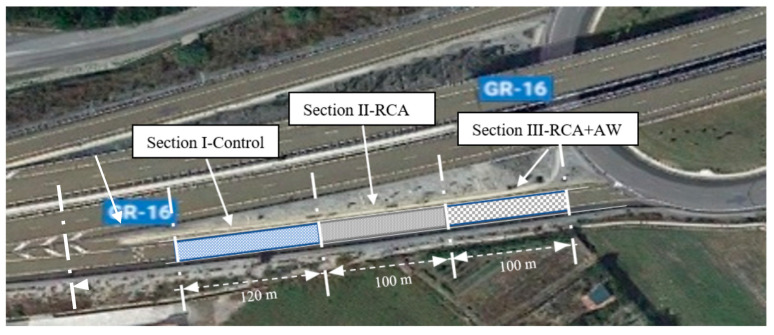
General view of the road section.

The experimental design involved studying and evaluating the physical, chemical, and mechanical behavior of the RCA and mixing RCA with AW for real-scale application in structural layers of the Gorgoracha–Puntalon road in Granada, Andalusia, Spain. Figure 1 shows the overview of the road sections.

The real-scale application was divided into three sections:

Section I-Control: From PK 0 + 000 to PK 0 + 120. The section includes AG in the road bases. AG is the conventional material used for the road base. This section will be used to compare the behavior of Section II and Section III manufactured with recycled materials.

Section II-RCA: From PK 0 + 120 to PK 0 + 220. The section is formed by RCA in the road base.

Section III-RCA + AW: From PK 0 + 220 to PK 0 + 320. It was designed with the mixture of RCA and 5% AW in the road base.

**Figure 2:**
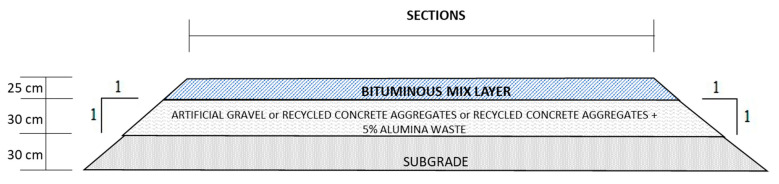
Structural layers of the road sections.

Figure 2 shows the structural layers of each section.

It is observed in Figure 2 that the pavement dimensioning maintains all the sections with the same thickness of the different projected layers. All of them are built as indicated by the Spanish Ministry 6.1-IC standard [33] “Pavement Section” of the Highway Instruction.

These sections guarantee structural capacity depending on the category of heavy traffic and type of esplanade. In accordance with the aforementioned standard, the thickness of these sections is checked by experience and verified using analytical methods.

## 3. Materials

Three types of materials were analyzed in this study. Artificial gravel (AG), recycled concrete aggregates (RCA), and alumina waste (AW). The latter material was used as an addition to RCA to evaluate its behavior as a treated granular material, using a mixture of 5% AW and 95% RCA called RCA + AW.

### 3.1. Artificial Gravel (AG)

AG is a mixture of aggregates, totally or partially crushed, in which the granulometry of all the elements that compose it is of a continuous type. It was obtained by crushing dolomite stone from Cantera de Vélez S.L, located in Vélez de Benaudalla (Granada, Spain). The most common use of this type of material is for the base of highways and roads, in the form of fillings of granular layers; therefore, it is ideal to be the reference sample.

### 3.2. Recycled Concrete Aggregates (RCA)

RCA was obtained from concrete construction waste, concrete products, mortar, and masonry factory parts from the Inertes Guhilar recycling plant in Granada (Spain). Table 1 shows the percentage by weight of each of the RCA components.

The RCA was composed of the following materials: ceramic, asphalt, natural aggregates, plaster, concrete, mortar, and impurities such as wood, glass, plastic, and metal. The components of the recycled aggregates were determined in accordance with EN 933-11:2009 [34]. The RCA was considered very pure because more than 90% was natural aggregate with and without concrete waste mortar. According to de Brito [35], due to its composition, it can be classified as RCA-I, being the best quality RCA according to its composition and physical and chemical properties.

Table 2 shows the information concerning the properties of AG and RCA materials tested in the laboratory according to the regulations indicated.

**Figure 3:**
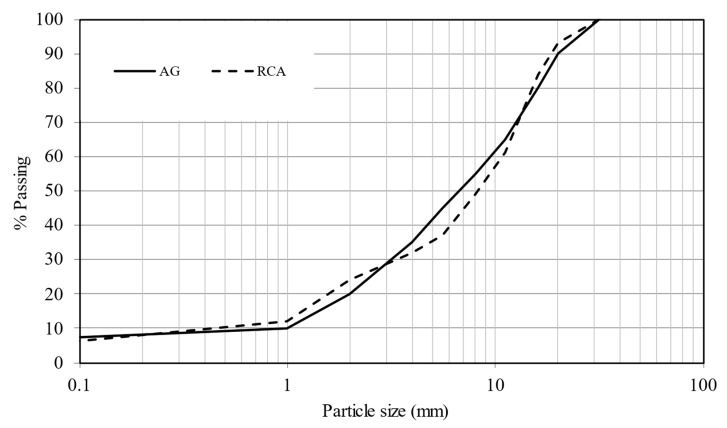
Particle size distribution curves limits of artificial gravel (AG) and recycled concrete aggregates (RCA).

All properties fulfilled the requirements of the Spanish General Technical Specification for Road Construction (PG-3) [34]. Important factors to consider in the RCA are the absorption content and the sulfate content. According to the proposal presented by de Brito [35] for the use of RA in road construction, RCA presented very good quality, with properties similar to those of a natural aggregate. The particle size distributions were analyzed according to the Spanish standard EN 933-1:2012 [42]. Figure 3 shows the particle size distribution curves. Both materials show a continuous distribution curve, which indicates a better degree of compaction. The percentage of fine fraction (<0.063 mm) was less than 10%.

### 3.3. Alumina Waste (AW)

The AW was a byproduct of a salt slag created during the aluminum scrap treatment process. The manager of this waste is Befesa Escorias Salinas Limited Society, Valladolid (Spain) This process occurs in three phases:

1. Crushing: The object of this treatment is, on the one hand, the extraction of the metallic aluminum, and, on the other hand, the reduction of the particle size to an optimum that ensures a perfect reaction of the dangerous components and a dissolution of the contained salts.
2. Dissolution-Reaction: The material (powder) obtained from grinding or received already ground is mixed with water to dissolve the salts. The dissolution is carried out with part of the condensate from the crystallization and with the filtrate from the alumina concentrate.
3. Crystallization: To separate the salts from the water contained in the brine obtained in the previous stage, the salts are separated by evaporation and subsequent condensation of the vapors. This produces a salt, a mixture of NaCl and KCl, and condensates that are reused in the process.

Table 3 shows the main components of the AW. A high content of Al_2_O_3_ (64.98%) is observed, which provides improvements in the mechanical properties according to Sua-Iam [43]. The silica content can produce expansiveness in the mixture with recycled aggregates [44]. Taking into account the value of 13.80% SiO_2_, the AW content in the mixture under study should be limited so as not to produce expansiveness.

## 4. Experimental Methods

### 4.1. Modified Proctor Test

The modified Proctor (MP) test is used to determine the dry density–moisture compaction ratio of the materials to be used in esplanades and firm layers, and as a reference for the quality control of the compaction on site. The ratio between dry density and compaction humidity is determined for a soil, for a compaction energy of 2.629 J/cm^3^, according to the standard EN 103-501:1994 [45].

### 4.2. California Bearing Ratio (CBR)

The California bearing ratio is used to evaluate the potential strength of subgrade, subbase, and base course material, including recycled materials for use in road and port pavements. The CBR value obtained in this test is an integral part of several flexible pavement design methods and is performed according to UNE 103-502:1995 [46], which describes the process for determining the soil resistance index CBR. This index is not an intrinsic material value but depends on the conditions of density, soil moisture, and overload to be applied when performing the test.

### 4.3. Accelerated Swelling Test for Soil Treated by Lime and/or Hydraulic Binder

This test involves the hydraulic setting of a sample of treated mixture, which is accelerated by applying special conservation conditions (20 °C and 100% moisture). At the end of a setting time period (maximum 14 days), the expansion of the specimen and the compressive strength are measured in accordance with regulations EN-13286-49:2004 [47].

Expansion is determined by the change in apparent volume of the test specimen as a function of time, first keeping it in a saturated atmosphere, and then submerging it in water at 40 °C.

## 5. Results and Discussion

The maximum dry densities and the optimal humidity were evaluated, as well as the bearing capacity using the California Bearing Ratio (CBR) test, of the AG, RCA, and the mixture of RCA with 5% AW (RCA + AW).

### 5.1. Modified Proctor Test/Moisture–Density Relationship

**Figure 4:**
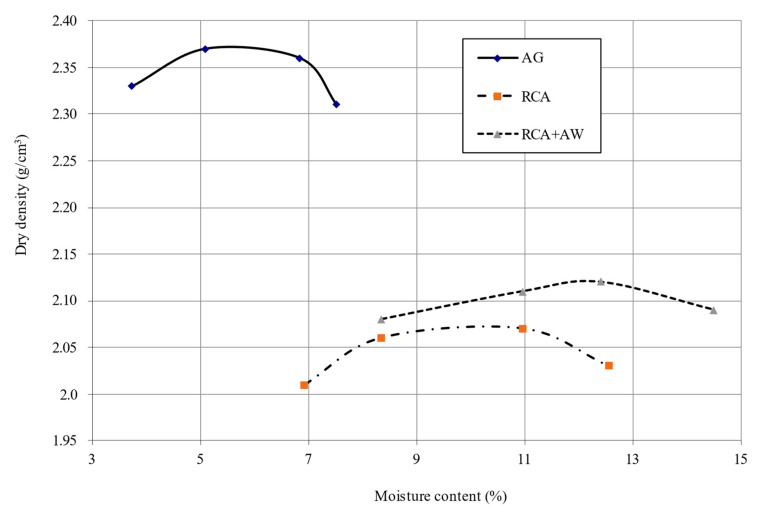
Moisture–dry density ratio of study specimens.

In the results of the MP test (Figure 4 and Table 4), the highest density was 2.37 g/cm^3^, and the lowest moisture was 6.01%. Both values correspond to the artificial gravel. The values in the RCA were density of 2.06 g/cm^3^ and moisture content of 10.20%. The values in RCA + AW were density of 2.12 g/cm^3^ and moisture content of 12.39%. Optimum moisture contents are higher for recycled materials, and the density is lower than that of AG because of the greater porosity of the material, according to other authors [28,48,49,50,51]. Moreover, the highest density value in RCA + AW with respect to the density value of RCA could be due to the fact that this mixture contains a high percentage of Al (34.39%, see Table 3), which, mixed with the sulfur content of RCA, causes a rapid hardening of the mixture [43,52].

RCA + AW shows a flat curve, which means that the density varies little with changes in humidity, and it is very important to consider this in its implementation to reach its maximum dry density with its optimum humidity.

### 5.2. California Bearing Ratio (CBR)

The CBR test was performed under different conditions: on the one hand, unsoaked, and on the other hand, soaked at 4 days, 28 days, 60 days and 180 days. The CBR test was performed with a 100% modified Proctor compaction process and the optimum water content of the material.

Table 5 shows the CBR values of AG, RCA, and RCA + AW at different ages under different conditions. Furthermore, Table 5 includes the increase in CBR over time. It is observed that the AG sample maintains the value of the load capacity over time, from a value of 100 in unsoaked conditions to 103 in 180-day soaked conditions. The RCA has the lowest CBR value, which ranges from 74 in unsoaked conditions to 98 in 180-day soaked conditions.

Other authors demonstrated that the CBR value of 30 is appropriate for granular materials in road layers [18]. It is observed that all the materials exceed this value.

The best result was obtained in the RCA + AW mixture. If we compare the CBR at 28 days for the RCA + AW mixtures with respect to the AG and RCA, the load capacity increases 10.52% (from 95 of AG to 105 of RCA + AW) and 28.57% (from 78 of RCA to 105 from RCA + AW), respectively.

In addition, an important value to highlight is the increase in load capacity that RCA and RCA + AW experience as the age of the mixture increases. This increase in both materials is probably due to the cementitious particles that they contain in their RCA composition. These values are in agreement with other authors. Poon [18] reported that the increase in CBR in RCA can be attributed to the unhydrated cement that constitutes RCA. The greater increase observed in RCA + AW could be due to the fact that this mixture contains a high percentage of Al (34.39%, see Table 3), which, mixed with the sulfur content of RCA, causes a rapid hardening of the mixture [32].

### 5.3. Accelerated Swelling Test for Soil Treated by Lime and/or Hydraulic Binder

The results are shown in Table 6.

As shown in Table 6, AG showed the lowest value of compressive strength with respect to the other two materials produced with RCA. These high values in RCA and RCA + AW are attributed to the self-cementing processes and the different chemical reactions within this type of material [18]. RCA + AW showed the best compression strength value, achieving a strength value 62% higher than the RCA strength value (0.55 in RCA and 0.89 in RCA + AW). However, the RCA + AW mixture showed greater swelling. This is due to the formation of ettringite with the hydration of cementitious particles, caused by the aluminum content of the alumina [53].

## 6. Real-Scale Application, Test Program, and Results

### 6.1. Compaction of Layers

We measured in situ density and soil moisture and aggregate through the use of nuclear equipment. Material density can be measured by direct transmission, backscatter, or backscatter/airspace methods. Measures for water content (moisture) are taken on the surface in backscatter mode, regardless of the mode used for density, and determined with nuclear density equipment, model Troxler 3430, according to ASTM D-6938 [54].

Between 10/12 measurements per section every 10 m were taken. The mean values of each material/section are included in Table 7.

Achieving good compaction is an influencing factor for mechanical behavior in unbound materials [17]. The compaction process was measured at the subgrade. The degree of compaction was calculated according to the optimum humidity and maximum density obtained in the modified Proctor test carried out in the laboratory and is shown in Table 7. The Spanish regulations [33] advise that the degree of compaction be between 95% and 98% of the value of the modified Proctor test of the reference. All materials met this standard. For example, in the AG, a compaction of 96% was achieved, since it goes from a density of 2.37 in the MP (Table 4) to a density of 2.28 g/cm^3^ achieved on site (Table 7), which means a 96% of the MP. The same occurs with the RCA and RCA + AW materials, which manage to reach densities on site that represent 97% and 95% of the MP, respectively. As shown in Table 7, the degree of compaction met the specifications of the current regulations [33]. Therefore, the sections were designed correctly.

### 6.2. Plate Bearing Test

The plate bearing test is a representative field-testing method that determines the bearing capacity factor considering the deformation characteristics of the subgrade. Plate bearing tests are used primarily in relation to temporary structures and working platforms: between the applied pressure and the displacements (pressure–displacement curve). According to the test procedure, a hydraulic device transfers pressure stepwise through a circular rigid plate onto the surface of the earth or rock half-space, until the displacement or pressure criterion is satisfied. A rigid plate with a diameter of 300 mm is the most commonly used.

These measurements allow to determine the compressibility module or coefficient. Two load cycles are carried out. The modulus of the first load cycle (Ev_1_) and the second modulus (Ev_2_) are defined by the following equation, valid for infinitely rigid circular plates, according to standard NLT-357/98 [55].

(1) $${\mathrm{Ev}}_{i}=1.5r\frac{\Delta \delta }{\Delta s}\;;\;i=1\;\mathrm{or}\;2$$

where ∆δ is the specific load difference transmitted by the plate between the loading steps to the ground or to the granular material in MPa; ∆S is the difference of plate seats when applying ∆P in mm; and r is the radius of the plate (298.5 mm).

Table 8 shows the compressibility modules Ev_1_ and Ev_2_. As can be seen, tests were carried out in three time periods (between October and November 2014), in order to analyze the evolution of the materials and mixtures under the action of the load. The subgrade had good capacity in all sections, obtaining the compressibility module in the second cycle (Ev_2_) well above the limits of the Spanish standard, which requires 180 MPa for a T1 traffic category [33].

The sections with the best performances were Sections II and III. It is observed that in addition to maintaining a good load capacity, this increased with time. Section III, made with RCA + AW, showed a notable increase due to the cementing properties of AW. It is mainly due to the phenomenon called rapid setting that occurs in materials such as RCA, with a high percentage of calcium sulfate, so that when AW is mixed (with a high Al value in its composition) with RCA, hydration of the calcium aluminates occurs, causing a rapid increase in resistance [56].

### 6.3. Deflection Measurements (FWD)

**Figure 5:**
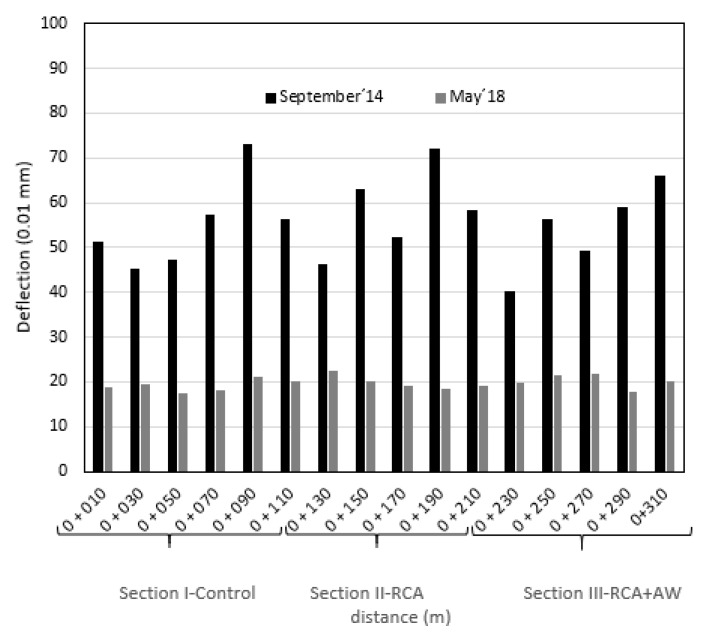
Deflection measurements of study sections.

The Dynatest HWD 8081 impact deflectometer (Dynatest, Madrid, Spain) was used for the development of the work according to General Technical Specifications for High-Performance Dynamic Monitoring Test. Using the FWD, surface data were taken every 20 m on two different dates, September 2014 and May 2018. Figure 5 shows the data obtained in each of the sections studied.

Deflection correction coefficients were applied for the humidity of the subgrade and pavement temperature, according to the corrections established in Regulation 6.3-IC of the Ministry of Development and standard NLT-338/07 [57].

In Figure 5, it is observed that all the sections improved with time, decreasing their deviations. In the case of Section I-Control, its average deviation in the September 2014 period was 58 × 10^−2^ mm, the same result as Section II-RCA (59 × 10^−2^ mm). However, the mixture of RCA + WA used in Section III shows an average deviation of 54 × 10^−2^ mm, being the one that behaves best in the short term. If we analyze the data obtained in the long term, May 2018, it is observed that the mean deflections in all the sections are similar, with an average deviation of 20 × 10^−2^ mm. Therefore, it is shown that industrial by-products such as RCA and RCA + AW equal the properties of raw materials such as AG.

## 7. Conclusions

In this study, the mechanical behaviors of RCA and the combination of RCA with alumina residues were evaluated and compared with artificial gravel (AG). To do this, an experimental section of road was built to evaluate its behavior under real traffic conditions. The following conclusions were drawn from this study:

- The mix of RCA and alumina used in this investigation had a higher load capacity at 28 days (CBR = 105) than did artificial gravel (CBR = 95), demonstrating the cementing capacity of RCA due to its non-hydrated cementitious particles and the elemental composition of alumina residues with a high percentage of Al.
- During the compaction process, the layers made with RCA and RCA + AW required higher humidity, as well as more demanding control, than do road layers made with natural materials.
- It was demonstrated that RCA, as well as the mixture of RCA with AW, are a viable option as raw materials for structural layers of unconsolidated roads, complying with the requirements imposed by Spanish regulations (PG3) for use as road material.
- The long-term evolution analyzed by the impact deviations showed that the modulus values increased significantly, showing similar patterns in all the sections, with a mechanical behavior of the recycled aggregates (and recycled aggregates with AW) samples equal to or better than that of the control sample.

In general, it is possible to use AW as a material mixed with RCA in the construction of road structural layers provided that appropriate and specific construction processes are adopted in their implementation, being an alternative for recycling and circular economy.

## Figures and Tables

**Table 1:** Constituents of recycled concrete aggregates (RCA) determined according to EN 933-11:2009 [34].

Composition	RCA
Ra (%) (Asphalt)	8.9
Rb (%) (Ceramics)	0.3
Rc (%) (Concrete and mortar)	42.1
Ru (Unbound aggregates)	48.5
X_1_ (%) (Others)	<0.1
X_2_ (%) (Gypsum)	<0.1

**Table 2:** Physical and chemical properties of artificial gravel (AG) and recycled concrete aggregates (RCA).

Properties	AG	RCA	Test Method
Water-soluble sulphate content (%)	0.05	0.33	EN 1744-1:2010 [36]
Acid-soluble sulphate content (%)	0.04	0.42	EN 1744-1:2010 [36]
Total sulphate content (SO_3_ %)	<0.01	0.47	EN 1744-1:2010 [36]
Organic matter (%)	0	0.68	UNE 103204:2019 [37]
Density-SSD (kg/m^3^)	-	-	EN 1097-6:2014 [38]
0–4 mm	2.77	2.48	-
4–31.5 mm	2.75	2.51	-
Water absorption (%)	-	-	EN 1097-6:2014 [38]
0–4 mm	0.61	5.45	-
4–31.5 mm	0.54	5.37	-
Flakiness index	13	13	EN 933-3:2012 [39]
Los Angeles ratio	25	35	EN 1097-2:2010 [40]
Sand equivalent	38	52	EN 933-8:2012 [41]

**Table 3:** Major components of alumina waste.

Concentration (g/100 g)							
Al	Si	Ca	Mg	Mn	Na	Fe	K
34.39	6.45	3.14	2.77	2.62	2.86	1.84	1.38
Al_2_O_3_	SiO_2_	CaO	MgO	MnO	Na_2_O	Fe_2_O_3_	K_2_O
64.98	13.80	4.39	4.59	3.38	3.86	2.63	1.66
**Loss on Ignition (g/100 g)**							
950 °C				150 °C			
11.37				3.49			

**Table 4:** Optimum moisture and maximum dry density of study specimens.

Materials	Optimum Moisture Content (%)	Maximum Dry Density (g/cm^3^)
AG	6.01	2.37
RCA	10.20	2.06
RCA + AW	12.39	2.12

**Table 5:** California Bearing Ratio (CBR) value and increase.

Test Conditions	CBR Value			Bearing Capacity Increase (%)		
-	AG	RCA	RCA + AW	AG	RCA	RCA + AW
Unsoaked	100	74	84	-	-	-
4-day soaked	94	60	68	-	-	-
28-day soaked	95	78	105	1.06	30.00	54.41
60-day soaked	98	91	117	4.25	51.60	72.05
180-day soaked	103	98	119	9.57	63.33	75.0

**Table 6:** Accelerated swelling of study specimens.

Materials	Initial Dry Density (g/cm^3^)	Compressive Strength (MPa)	Swelling after 7 Days of Soaking (%)
AG	2.37	0.04	2.6
RCA	2.16	0.55	2.3
RCA + AW	2.15	0.89	3.1

**Table 7:** In situ assessments of density and moisture of study specimens.

Properties	AG Section I	RCA Section II	RCA + AW Section III
Density (g/cm^3^)	-	-	-
Mean	2.28	1.99	2.01
Compaction (%)	-	-	-
Mean	96% of MP *	97% of MP	95% of MP
Moisture content (%)	-	-	-
Mean	5.50	9.20	11.34

* MP: Modified Proctor.

**Table 8:** Value of plate bearing test for study sections.

Sections	First Load Plate		Second Load Plate		Third Load Plate		Middle Value	
-	Ev_1_ (MPa)	Ev_2_ (MPa)	Ev_1_ (MPa)	Ev_2_ (MPa)	Ev_1_ (MPa)	Ev_2_ (MPa)	Ev_1_ (MPa)	Ev_2_ (MPa)
Section I (Control)	180	900	214	750	225	1500	214	900
Section II (RCA)	132	642	107	750	74	643	107	643
Section III (RCA + AW)	115	409	113	750	104	1125	113	750

## Data Availability

The data are contained in the article. The data presented in this study are available in the journal publication materials [title: Feasible Use of Recycled Concrete Aggregates with Alumina Waste in Road Construction].
